# Gabapentin Enhances the Morphine Anti-Nociceptive Effect in Neuropathic Pain via the Interleukin-10-Heme Oxygenase-1 Signalling Pathway in Rats

**DOI:** 10.1007/s12031-014-0262-2

**Published:** 2014-02-27

**Authors:** Yu-Hua Bao, Quan-Hong Zhou, Rui Chen, Hao Xu, Lu-Lu Zeng, Xin Zhang, Wei Jiang, Dong-Ping Du

**Affiliations:** 1 Pain Management Center, Shanghai Six People’s Hospital, Shanghai Jiaotong University, No. 600 Yishan Road, Shanghai, 200233 People’s Republic of China; 2 Department of Anesthesiology, Shanghai Six People’s Hospital, Shanghai Jiaotong University, No. 600,Yishan Road, Shanghai, 200233 People’s Republic of China

**Keywords:** Morphine tolerance, Neuropathic pain, Gabapentin, Interleukin(IL)-10, Heme-oxygenase (HO)-1, Inflammatory cytokine

## Abstract

In the present study, we investigated the anti-inflammatory mechanisms by which gabapentin enhances morphine anti-nociceptive effect in neuropathic pain in rats and the interaction between the anti-nociceptive effects of gabapentin on morphine and the interleukin (IL)-10-heme-oxygenase (HO)-1 signal pathway in a rat model of neuropathic pain. The neuropathic pain model was induced via a left L5/6 spinal nerve ligation (SNL) in rats. The anti-nociceptive effect of gabapentin and IL-10 on morphine was examined over a 7-day period, and the effects of the anti-IL-10 and HO-1 inhibitor zinc protoporphyrin (ZnPP) on gabapentin/morphine co-injection were assessed. Drug administration was given over 7 days, and on day 8, both anti-inflammatory cytokine IL-10, a stress-induced protein HO-1 and pro-inflammatory cytokines IL-1β, IL-6 and TNF-α were measured. Gabapentin attenuated morphine tolerance over 7 days of co-administration, and reduced the expression of pro-inflammatory cytokines but increased IL-10 and HO-1 expression. The effect of gabapentin on morphine was partially blocked using the anti-IL-10 antibody or the HO-1 inhibitor zinc protoporphyrin. Our findings indicated that the anti-nociceptive effects of gabapentin on morphine might be caused by activation of the IL-10-HO-1 signalling pathway, which resulted in the inhibition of the expression of pro-inflammatory cytokines in neuropathic pain in the rat spinal cord.

## Introduction

Neuropathic pain remains a serious clinical problem due to the lack of efficacy of available therapeutic modalities. Using opioids to control neuropathic pain has proven to be effective in animals and humans, challenges with analgesic tolerance have limited its use. The mechanism underlying opioid tolerance is complex and poorly understood particularly under conditions of neuropathic pain. Thus, a deeper understanding of the mechanisms underlying morphine tolerance under conditions of neuropathic pain may contribute to the development of better analgesic treatments for pain.

Previous studies have demonstrated that both pro-inflammatory and anti-inflammatory cytokines are involved in the establishment and maintenance of morphine tolerance and neuropathic pain (Johnston et al. 2004; Schafers and Sommer 2007; Uceyler and Sommer 2008; Shen et al. 2011). Opioids-induced hyperalgesia is observed in tolerance; it is similar to the symptoms observed in neuropathic pain, where opioids also have a limited analgesic effect (Mika et al. 2004; Narita et al. 2013). Numerous studies have indicated that neuropathic pain results in reduced morphine efficacy and a more rapid development of morphine tolerance (Ossipov et al. 1995; Mayer et al. 1999; Mika et al. 2004; Przewlocki and Przewlocka 2005). It has been shown that the suppression of glial activation, which in turn, inhibits pro-inflammatory cytokine synthesis, can improve morphine efficacy in treating neuropathic pain (Song and Zhao 2001; Raghavendra et al. 2002; Watkins et al. 2007). IL-10 is a powerful anti-inflammatory cytokine with a wide spectrum of biological effects. A previous study has shown that the anti-inflammatory cytokine IL-10 exhibits anti-allodynic or anti-hyperalgesic effects by inhibiting the release of the pro-inflammatory cytokines TNF-α and IL-1β via the peritoneal macrophages (Laughlin et al. 2000; Wang et al. 2012). IL-10 not only demonstrates an anti-nociceptive effect in neuropathic pain rats with spared nerve injury but also plays a pivotal role in morphine tolerance, via its regulation of the production of pro-inflammatory cytokines and nerve growth factor. Several studies have demonstrated that acute administration of 5, 10 and 20 mg/kg morphine decreased the expression of IL-10 in a dose-dependent manner (Limiroli et al. 2002). In addition, daily intrathecal (i.t.) injection of 1 μg of rrIL-10 significantly preserved the anti-nociceptive effects of morphine in chronic morphine-infused rats. Moreover, gene therapy with an adenoviral vector encoding IL-10 potentiated acute morphine analgesia and attenuated the development of morphine tolerance (Sacerdote 2003; Lin et al. 2010). Additional studies have demonstrated that the powerful anti-inflammatory effect of IL-10 is associated with heme-oxygenase (HO)-1. In murine macrophages, IL-10 induces the expression of HO-1, a stress-induced protein with a potent anti-inflammatory effect, which enhances the anti-inflammatory capacity of IL-10, and anti-10 antibody reversed the amitriptyline-induced upregulation of HO-1 expression in morphine-tolerant rats (Drechsler et al. 2006; Tai et al. 2009).

Gabapentin is also used as an anti-convulsant drug and is now widely becoming accepted as an alternative treatment for various types of neuropathic and inflammatory pain (Boroujerdi et al. 2011; Thomas and Farquhar-Smith 2011; Yeh et al. 2011). Gabapentin also has a well-established role in the attenuation of morphine tolerance. Previous preclinical studies have demonstrated that gabapentin increases the anti-nociceptive effect of morphine in an acute model of nociception and in a visceral nociception model (Meymandi and Sepehri 2008). An electrophysiological study showed that this combination inhibited evoked dorsal horn neuronal responses in a rat model of neuropathy (Matthews and Dickenson 2002). However, the mechanism of the gabapentin enhancement of morphine-induced anti-nociceptive effect in a neuropathic pain model is not very well understood. In our previous studies, we found that gabapentin-attenuated morphine tolerance is associated with the upregulation of spinal anti-inflammatory cytokine IL-10 and downregulation of pro-inflammatory cytokines TNF-α, IL-1β and IL-6. Byung-Sang et al. found that the anti-nociceptive effects of gabapentin might be caused by an upregulation of IL-10 expression, which results in the inhibited expression of pro-inflammatory cytokines in neuropathic pain rats (Lee et al. 2013). Thus, inflammatory mechanisms play an important role in the gabapentin rescue of the anti-nociceptive effects of morphine.

In our present study, we examined the potential mechanism underlying the effect of gabapentin on the inhibition of spinal cord neuroinflammation. We hypothesised that gabapentin enhanced the morphine anti-nociceptive effect via IL-10 and its downstream HO-1 signal transduction pathway to inhibit pro-inflammatory cytokine expression in neuropathic pain rats. Taken together, our results provide insight for the role of gabapentin in clinical pain management, particularly in patients who suffer from neuropathic pain and require long-term opioid treatment.

## Materials and Methods

### Animals

The experiments were performed on male Sprague–Dawley rats (200–250 g) provided by the Shanghai Laboratory Animal Center at the Chinese Academy of Science (Shanghai, China) and were approved by the Animal Care and Use Committee of the Medical School of the Shanghai Jiao Tong University. The rats were individually housed after i.t. catheterisation. Food and water were available ad libitum. The temperature and relative humidity were maintained at 23 ± 1 °C and 50–55 %, respectively. The animal room was artificially maintained on a 12-h light/12-h dark cycle (lights on 08:00–20:00 h).

### Intrathecal Catheterisation

For i.t. drug administration, the rats were anesthetised with pentobarbital (50 mg/kg, intraperitoneal) using a PE-10 i.t. catheter (Becton Dickinson, Sparks, MD, USA), which was inserted via an incision in the atalanto-occipital membrane of the cisterna magna such that the tip of the catheter is placed at the lumbar enlargement. The bead end was cut each time prior to i.t. injection and was fixed for the next use. The rats were allowed to rest for 1 day after catheterisation, and lidocaine (2 %, 10 μl) was subsequently injected through the catheter to test its position. Only the rats that showed immediate paralysis on both sides (30 s) after lidocaine injection were kept in the study. The drugs were injected through the i.t. catheter. After a recovery period of 7 days, the animals without locomotion deficits were subjected to the neuropathic pain model (*n* = 6 each group).

### Neuropathic Pain Models

One week after i.t. injection, ligation of the L5/6 spinal nerves in rats was used as an experimental model of neuropathic pain in this study, which was performed according to the method previously described by Kim and Chung (1992). All animals were anaesthetised with sodium pentobarbital (50 mg/kg, intraperitoneally). A dorsal midline incision was made from L3 to S2 and the left L5 and L6 spinal nerve were isolated and tightly ligated with 4-0 silk sutures. The incision was then closed in two layers. The sham surgery involved the sham procedure but without nerve ligation. Only animals presenting with confirmed tactile allodynia and exhibiting no motor weakness were used for this experiment. After surgery, each rat was injected intraperitoneally with penicillin 0.2 million units per day for 6 consecutive days (*n* = 6 each group).

### Drugs and Treatment

All animal experiments were performed on day 7 after the spinal nerve ligation (SNL) model established. Recombinant rat IL-10 (rrIL-10; 1, 2 and 5 μg in 10 μl; Cell Science, Canton, MA, USA), anti-IL-10 antibody (1, 5 and 10 μg in 10 μl; Abcam), HO-1 antagonist zinc protoporphyrin (ZnPP; 24 μg in 10 μl; Tocris Cookson, Bristol, UK), morphine (30 μg in 5 μl; Yichang, China) and gabapentin (25 μg in 5 μl, Sigma). All drugs were dissolved in 0.9 % saline and were administered twice a day for 7 days via a PE-10 injection using a microinjector, followed by a 10-μl physical flush. When two or three drugs were co-administered, there was a 1–2 μl air in the syringe to separate them. Doses of gabapentin, recombinant rat IL-10, anti-IL-10 antibody and Znpp were selected on the basis of previous studies and our preliminary studies. All of these drugs when administered alone had no effect on neuropathic pain in rats. The doses of morphine were chosen on the basis of our preliminary work, which demonstrated that 30 μg had an anti-nociceptive effect on neuropathic pain rats. Animals were randomly placed into either the drug or vehicle groups according to baseline testing, and the testing was performed in a blind manner. Behavioural tests were performed at 30 min after each daily drug injections. All rats in each group were sacrificed after the 7-day course of treatment for tissue extraction (*n* = 6 each group).

### Behavioural Analysis

Seven days after establishing the neuropathic pain model, behavioural testing was performed. All rats were habituated in the experimental arena. Nociceptive testing was initially performed using the tail–flick test and then with the paw-withdrawal test. Animal behavioural testing was measured 30 min after the first drug was administered each day. The investigator was blind to the treatment condition of the rats.

### Tail–Flick Test

Thermal hyperalgesia was assessed using the tail–flick test according to the method described by Vanderah et al. This test was performed in a warm water (52.5 ± 0.5 °C) tail–flick apparatus (Shanghai Thermo-stat Factory, Shanghai, China). The nociceptive endpoints were the characteristic withdrawal of the tail from the warm water. To avoid tissue damage, the heat stimulus was discontinued after 10 s (cut-off latency). The baseline latency was obtained before any drugs were used. The following response latencies were determined at 30 min after the administration of the drug(s). Tail–flick values were converted to a maximum percent effect (MPE): MPE = 100 × [(post-drug latency - baseline) / (cut-off latency - baseline)] (*n* = 6 each group).

### Von Frey Test

Mechanical hyperalgesia in rats was measured using a series of calibrated nylon Von Frey filaments (Stoelting, Wood Dale, IL, USA) ranging from 0.4 to 26 g. The Von Frey filaments were applied in ascending order to the mid-plantar surface of the operated hind paw through the mesh floor. Brisk withdrawal or paw flinching were considered positive responses. Each probe was applied to the foot until it started to bend. The paw withdrawal threshold (PWT) was calculated using the ‘up–down’ method, and these data were analysed using the nonparametric method of Dixon as described by Chaplan et al. (1994). An examiner who was blind to the treatment groups performed all of the behavioural tests (*n* = 6 each group).

### Enzyme-Linked Immunosorbent Assay

On day 8, all of the rats were sacrificed following an overdose of pentobarbital anaesthesia. The L4–L6 left-half of the spinal cord was immediately removed and stored at -80 °C prior to further treatment. Frozen samples were directly homogenised in a buffer in the presence of protease inhibitors (Sigma). After centrifugation at 13,000 rpm for 20 min, the supernatant was obtained for IL-1, IL-6, TNF-α and IL-10 protein analysis. The Bradford protein assay was used to determine the total protein concentration. Commercially available enzyme-linked immunosorbent assay (ELISA) kits were used to assess the cytokine proteins (R&D Systems, Minneapolis, MN, USA; sensitivity: 5 pg/ml). ELISA microplates were analysed using a Victor3 V multilabel counter (1420, Perkin-Elmer, Boston, MA, USA), and data were standardised as picograms of TNF-α, IL-1β, IL-6 and IL-10 per 200 μg of total supernatant protein. The concentration of each target cytokine was determined based on an appropriate set of internal standard curves using recombinant rat cytokines (*n* = 6 each group).

### Western Blotting Analyses

Frozen samples were directly homogenised in a buffer (Beyotime, Nanjing, Jiangsu, China) in the presence of protease inhibitors (Sigma-Aldrich, USA). After centrifugation at 12,000 rpm for 30 min at 4 °C, the supernatant was obtained for Western blotting analysis. BCA (Pierce, Thermo Fisher Scientific, Waltha, MA) methods were used to assay the protein concentration. The total protein (50 μg) was loaded onto a 10 % sodium dodecyl sulphate (SDS) polyacrylamide gel and transferred onto polyvinylidene difluoride (PVDF) membranes (Millipore, Billerica, MA, USA) for Western blotting analyses. The membranes were blocked with 10 % non-fat milk in Tris-buffered saline (TBS) for 2 h at room temperature (RT) and incubated with antibodies overnight at 4 °C with monoclonal mouse anti-HO-1 (1:2000, Abcam) antibody or goat anti-β actin (1:10000, Sigma) primary antibodies. The membranes were washed with TBS containing 1 % Tween and incubated with horseradish peroxidase-conjugated secondary antibodies (1:2,500; donkey anti-rabbit; Cell Signaling, USA) for 1 h at room temperature. Finally, the proteins were detected using electrochemiluminescence reagents (Pierce Biotechnology, Rockford, IL, USA) and visualised by film exposure. The protein band density was quantified using densitometric scanning. All Western blotting analyses were performed at least three times, and parallel results were recorded (*n* = 6 each group).

### Statistical Analysis

All values were presented as the mean ± standard error (SEM) and analysed using Graphpad Prism software. The SNL model of Von Frey filament test was determined using nonlinear regression analysis followed by one-way analysis of variance (ANOVA) analysis. For 7 days, behavioural tests were analysed using a two-way repeated-measures analysis of variance (treatment by time). For statistical analysis, immunoreactivity and cytokine data were analysed using one-way ANOVA followed by multiple comparisons using the Student–Newman–Keuls post hoc test. *p* < 0.05 was considered statistically significant.

## Results

### The Established SNL Model

**Fig. 1:**
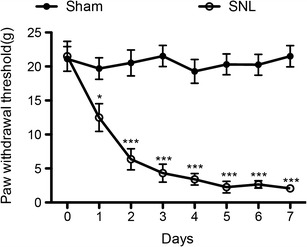
Time course of paw withdrawal threshold after spinal nerve ligation (*SNL*) and sham surgery over a 7-day period. All data points are expressed as the mean ± SEM (*n* = 6, each). *p < 0.05, ^***^ *p* < 0.001 compared with sham group

As shown in Fig. 1, 1 week after SNL, the mechanical withdrawal threshold of the hind paw ipsilateral to the nerve injury was significantly decreased in SNL rats (*p* < 0.001) compared to the sham-operated animals. In addition, no difference in the withdrawal threshold was observed in the contralateral hind paw. These results suggested that the neuropathic pain model with monolateral mechanical allodynia has been successfully established and was consistent with previous reports.

### Effects of Gabapentin and Anti-IL-10 Antibody on Morphine Administration in SNL Rats

**Fig. 2:**
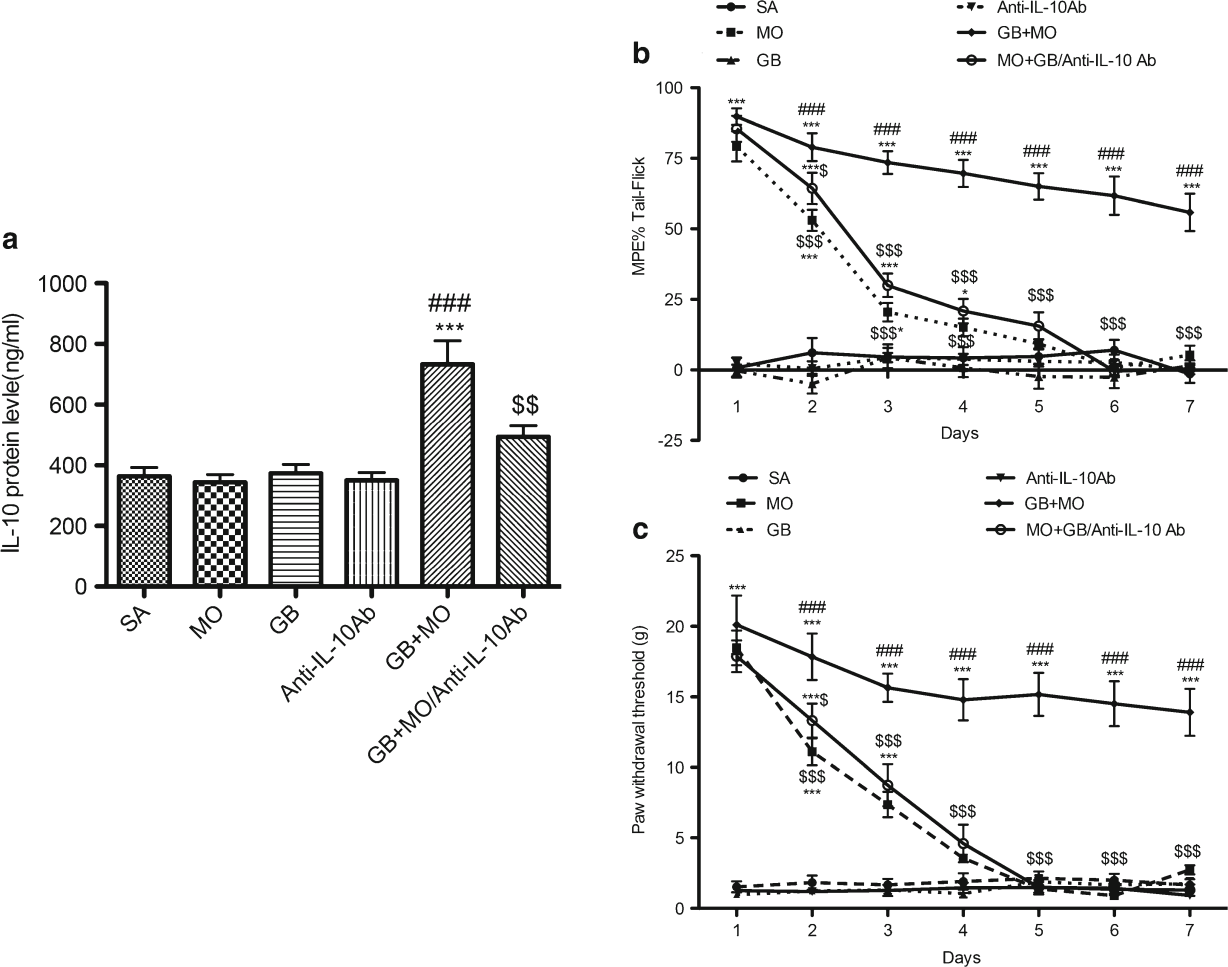
Il-10 anti-body abolished the effect of gabapentin on morphine in SNL exposed rats. **a** IL-10 protein levels were measured in the experimental group. Co-administration of gabapentin (25 μg) with morphine (30 μg) for 7 days twice daily significantly increased IL-10 expression in rats; anti-IL-10 antibody (10 μg) neutralised IL-10 expression induced by this co-administration; gabapentin delayed morphine tolerance development in SNL-exposed rats, as measured using the tail–flick (**b**) and Von Frey (**c**) tests. The maximum anti-nociceptive effect of morphine occurred on days 1–3. From days 4 to 7, there was no difference between the morphine and saline groups. When gabapentin was co-administered with morphine and prevented morphine tolerance. The anti-IL-10 antibody partially reversed the potentiating effect of gabapentin on morphine in SNL rats (*n* = 6, each). ^***^ *p* < 0.001 compared with the saline group; ^###^ *p* < 0.001 compared with the morphine group; ^$^ *p* < 0.05, ^$$^ *p* < 0.01, ^$$$^ *p* < 0.001 compared with the gabapentin/morphine group

In SNL rats, chronic morphine tolerance was attenuated by co-administration of gabapentin with morphine twice a day for 7 days in the behavioural tests (Fig. 2b, c). The efficacy of morphine was diminished after 4 days of injection, but the efficacy was maintained on day 7 when morphine was co-administered with gabapentin. The anti-IL-10 antibody abolished the effect of gabapentin on morphine. Moreover, there was no difference in anti-nociception effect in the saline group or the gabapentin and anti-IL-10 antibody-treated animals.

On day 8, analysis of the lumbar spinal cord homogenates demonstrated that an i.t. daily injection of anti-IL-10 antibody (10 μg) alone did not affect IL-10 expression compared with the saline-injected rats (Fig. 2a). Furthermore, co-injection of gabapentin (25 μg)/morphine (30 μg) clearly increased the anti-inflammatory cytokine IL-10 protein levels compared to that in the saline-, gabapentin- and morphine-injected rats. Daily injection of anti-IL-10 antibody significantly neutralised the effect of gabapentin in the morphine-injected rats.

### Effects of IL-10 Regarding the Improvement of the Anti-Nociceptive Effect of Morphine in Neuropathic Pain Rats

**Fig. 3:**
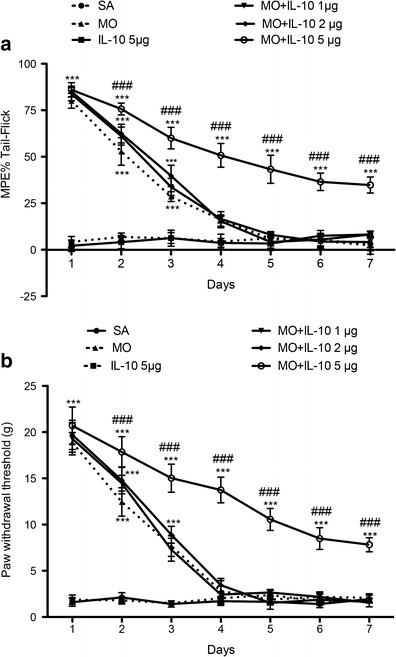
Effects of IL-10 on the anti-nociceptive effects of morphine in SNL rats. Time-course of the tail–flick (**a**) and Von Frey filament (**b**) tests after 7 days of daily injection of various doses (1, 2 or 5 μg) of recombinant rat IL-10 (rrIL-10) combined with continuous i.t. morphine injection. A large dose injection of rrIL-10 (5 μg) with morphine for 7 days produced a significantly anti-nociceptive effect, whilst rrIL-10 (5 μg) administered alone did not produce an anti-nociceptive effect in SNL rats (*n* = 6, each). ^***^ *p* < 0.001 compared with the saline group; ^###^ *p* < 0.001 compared with the morphine group

Recombinant rat IL-10 (rrIL-10) was used to investigate the effect of IL-10 in the attenuation of the development of morphine tolerance. Daily intrathecally injections of rrIL-10 (5 μg) alone did not produce any anti-nociceptive effect via the 7-day treatment compared with the saline group (Fig. 3a, b). The anti-nociceptive effect of continuous morphine injection was partially maintained via a daily intrathecal rrIL-10 (5 μg) injection during tolerance induction but not by an injection of lower doses of rrIL-10 (1 or 2 μg) compared with the morphine group. These data demonstrated that IL-10 attenuated morphine tolerance in neuropathic pain rats.

### Anti-IL-10 Antibody-Induced Effects of HO-1 Expression Are Dose-Dependent

**Fig. 4:**
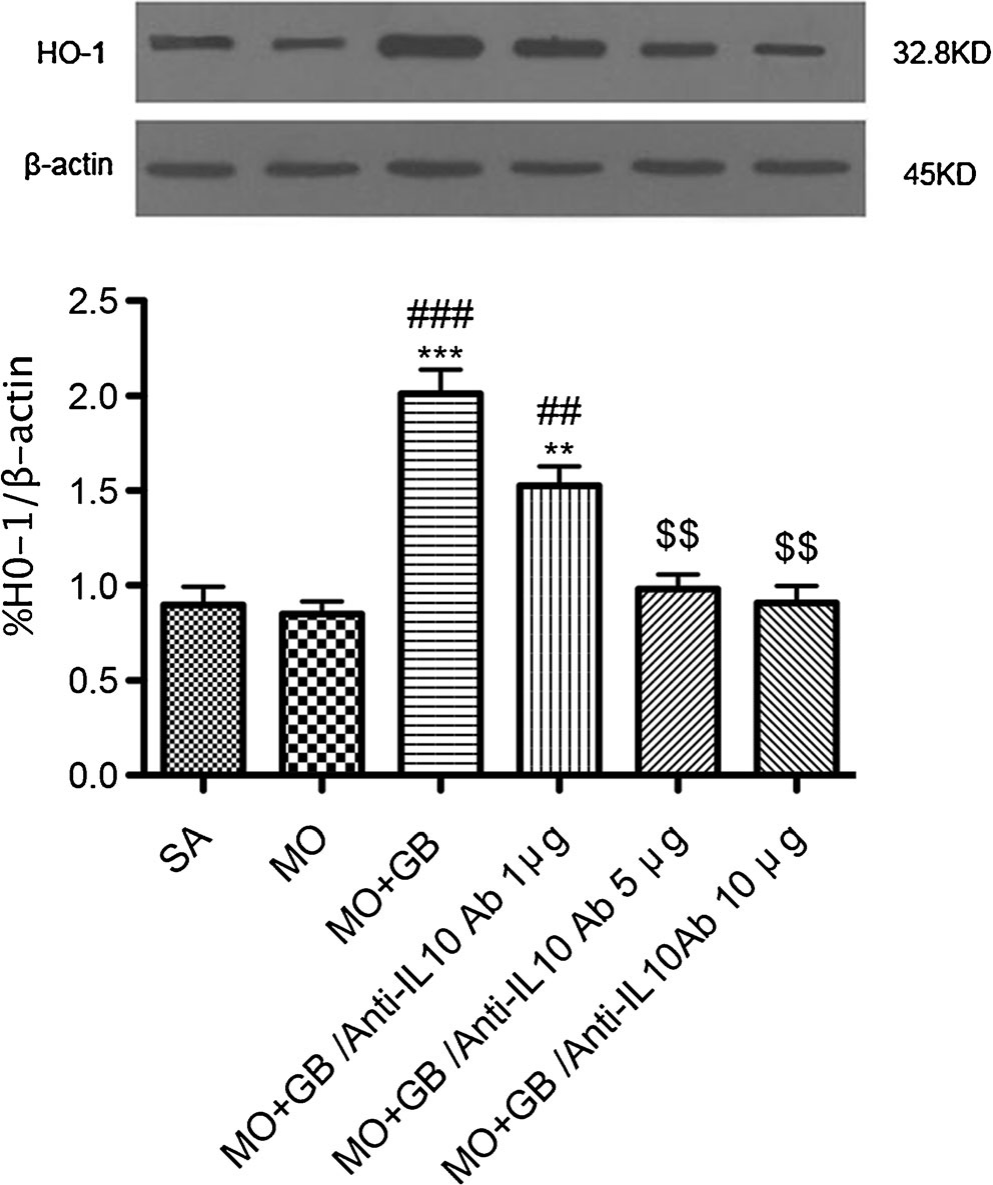
Anti-IL-10 antibody dose-dependently reversed gabapentin-induced heme oxygenase-1 (*HO*-*1*) expression in gabapentin/morphine co-injected SNL rats. Western blotting analyses of HO-1 in the spinal cord dorsal horn of the various treatment groups; β-actin was used as the internal control. Importantly, the upregulated HO-1 protein observed in animals co-administered with gabapentin and morphine was suppressed by different concentrations of anti-IL-10 antibody (*n* = 6, each). ^**^ *p* < 0.01, ^***^ *p* < 0.001 compared with the saline group; ^##^ *p* < 0.01, ^###^ *p* < 0.001 compared with the morphine group; ^$$^ *p* < 0.01 compared with the gabapentin/morphine group

Co-injection of gabapentin and morphine for 7 days significantly increased HO-1 expression compared with the morphine-treated animal group (Fig. 4). In addition, daily i.t. injection of different doses of anti-IL-10 antibody for 7 days dose-dependently reversed the gabapentin-induced upregulation of HO-1 expression. These results suggested that IL-10 is involved in the increase in HO-1 expression in gabapentin/morphine–injected neuropathic pain rats.

### Role of the HO-1 Inhibitor ZnPP in Reversing Gabapentin Enhancement of Morphine Anti-Nociceptive Effects in Neuropathic Pain Rats

**Fig. 5:**
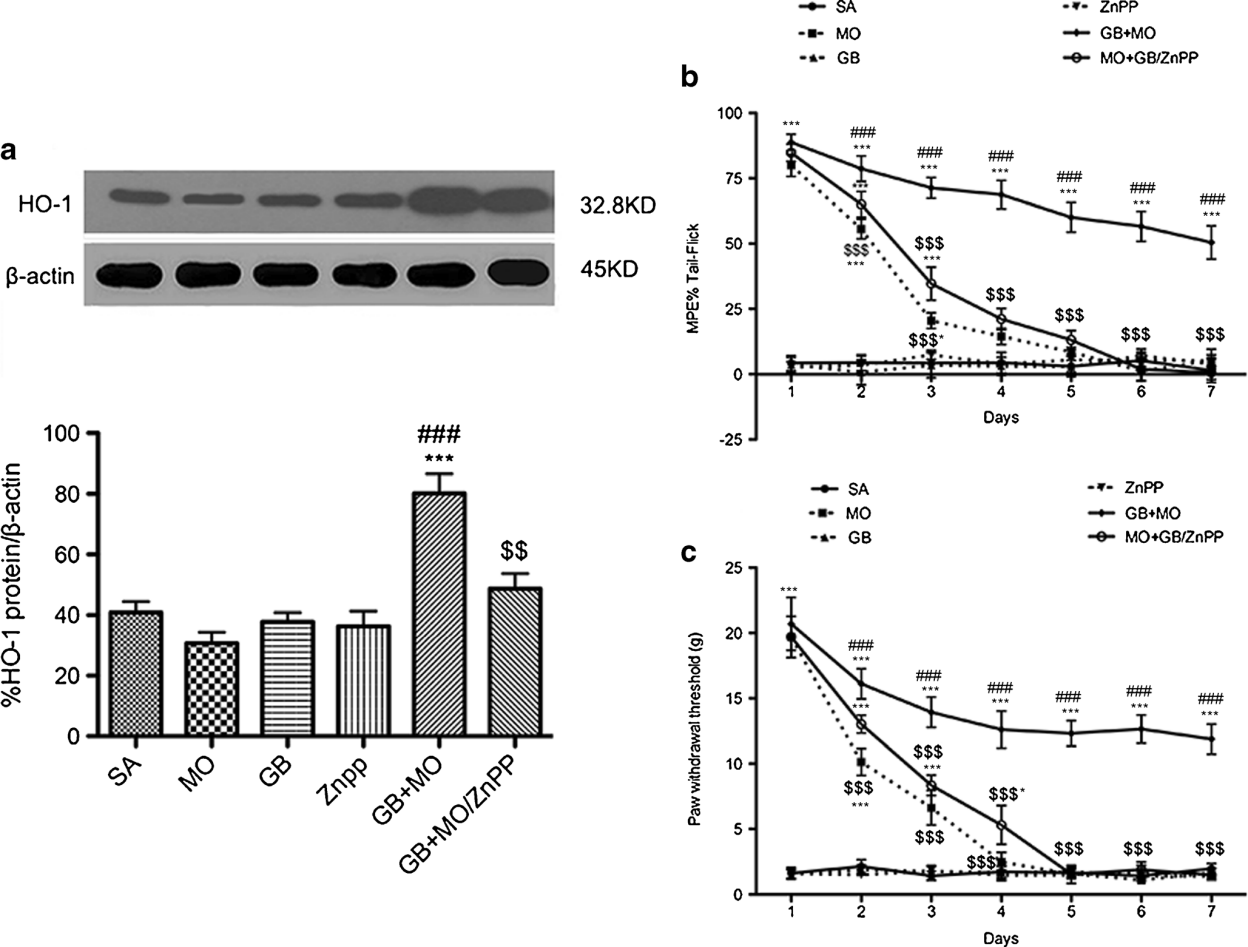
HO-1 antagonist zinc protoporphyrin reversed the gabapentin-induced upregulation of heme oxygenase-1 (*HO*-*1*) expression and potentiated the anti-nociceptive effect of morphine in gabapentin/morphine-co-injected SNL rats. **a** Western blotting analysis of HO-1 in the rat spinal dorsal horns. β-actin was used as the loading control. The relative band densities for HO-1 after different treatments. Time course of tail–flick test (**b**) and Von Frey latencies (**c**) in SNL rats. The maximum anti-nociceptive effect of morphine occurred on days 1–2. From days 4–7, there was no difference between the morphine and saline-treated animals. When gabapentin was co-administered with morphine, it prevented morphine tolerance. In addition, ZnPP partially reversed the potentiating effect of gabapentin on morphine in SNL rats (*n* = 6, each). ^*^ *p* < 0.05, ^***^ *p* < 0.001 compared with the saline group; ^###^ *p* < 0.001 compared with the morphine-injected group; ^$^ *p* < 0.05, ^$$^ *p* < 0.01 ^$$$^ *p* < 0.001 compared with the gabapentin/morphine-injected group

Daily i.t. injection of the HO-1 antagonist zinc protoporphyrin (ZnPP) with gabapentin/morphine in neuropathic pain rats for 7 days reversed the upregulation of HO-1 expression in gabapentin/morphine injected rats (Fig. 5a). In addition, it blocked the maintenance of morphine’s anti-nociceptive effect by gabapentin in SNL rats. Consistent with these results, the maximal anti-nociceptive effect of morphine was observed on day 1, and the maximal tolerance was observed on day 4 in morphine-injected rats. Co-administration of ZnPP with gabapentin plus morphine blocked the maintenance of morphine’s anti-nociceptive effect by gabapentin and decreased the expression of HO-1 in spinal cords from day 3 to day 7 (Fig. 5b, c).

### Inhibition of IL-10 Expression and HO-1 Expression Reverses the Suppressive Effects of Gabapentin on Cytokine Production in Morphine-Injected SNL Rats

**Fig. 6:**
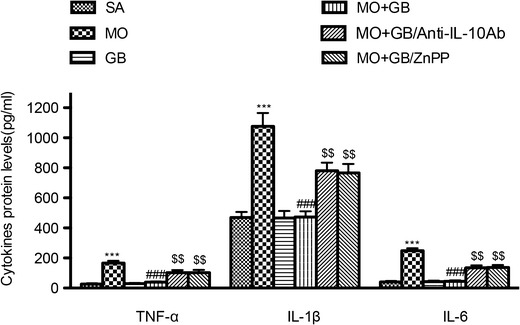
Protein levels for tumor necrosis factor-α (*TNF*-α), interleukin (*IL*)-1β and IL-6 were measured in the different groups. Anti-interleukin-10 antibody and heme oxygenase-1 antagonist zinc protoporphyrin reversed the suppressive effect of gabapentin on pro-inflammatory cytokine production (*n* = 6, each). ^***^ *p* < 0.001 compared with the saline-injected group; ^###^ *p* < 0.001 compared with the morphine-injected group; ^$$^ *p* < 0.01 compared with the saline, morphine and gabapentin/morphine-injected group

As shown in Fig. 6, protein levels for the pro-inflammatory cytokines IL-1β, IL-6 and TNF-α were significantly increased in morphine-injected rats compared with the saline-injected rats, and this effect was significantly attenuated by gabapentin co-injection. Furthermore, injection of anti-IL-10 antibody and the HO-1 antagonist ZnPP significantly abolished the suppressive effect of gabapentin on pro-inflammatory cytokine production in morphine-injected SNL rats.

## Discussion

In the present study, over 7 days, chronic morphine injection produced anti-nociceptive tolerance in an SNL rat model of neuropathic pain. In addition, gabapentin enhanced the anti-nociceptive effect of morphine. This effect could be explained, at least in part, by gabapentin-induced downregulation of pro-inflammatory cytokine TNF-α, IL-1β and IL-6 expression and upregulation of anti-inflammatory cytokine IL-10 expression in the rat spinal cord. Improvement of the analgesic effects of morphine by gabapentin was partially blocked by co-administration of anti-IL-10 antibody and the HO-1 antagonist ZnPP. Neutralisation of the IL-10 antibody reversed the gabapentin-induced increase in HO-1 expression and inhibition of pro-inflammatory cytokines expression, suggesting that the HO-1 pathway is involved in gabapentin-mediated induction of IL-10 expression. These results suggested that the suppressive effect of gabapentin co-injection on pro-inflammatory cytokine production in morphine-injected rats may act via increasing IL-10 and HO-1 protein expression. Several studies have shown that the mRNA and protein levels of the pro-inflammatory cytokines TNF-α, IL-1β and IL-6 increased following chronic morphine injection and neuropathic pain (Rittner et al. 2005). The release of pro-inflammatory cytokines is a consequence of chronic morphine exposure, which resulted in the reduction of the anti-nociceptive effect of morphine. Our results suggest that the gabapentin enhancement of the anti-nociceptive effect of morphine in the neuropathic pain model in rats is related to an inhibition of spinal inflammation.

IL-10 was first described as an inhibitor of cytokine synthesis, and it was subsequently demonstrated that IL-10 attenuates nociceptive effects by inhibiting spinal glia activation and the production of pro-inflammatory cytokines in an animal model (Niu et al. 2012). Furthermore, IL-10 abolishes the mechanical hyperalgesia induced by carrageenan administration in the hind paw of rats. An IL-10-encoding adenoviral vector not only attenuated the development of tolerance, hyperalgesia and allodynia, it also potentiated the analgesia effect of morphine (Milligan et al. 2005). Chronic morphine treatment has been shown to not alter IL-10 mRNA expression levels and to even reduce lippolysaccharide (LPS)-induced IL-10 secretion (Nelson and Lysle 2001; Messmer et al. 2006). In our study, an increase in IL-10 protein levels was observed in the spinal cord of gabapentin/morphine co-administered neuropathic pain rats. Moreover, daily IL-10 (5 μg) i.t. injections significantly improved the anti-nociceptive effect of morphine. Taken together, these results strongly indicated that the inhibitory effect of IL-10 activation and cytokine production are responsible for the neuroinflammation induced by chronic morphine injection in neuropathic pain rats. Consistent with this finding, a neutralising anti-IL-10 antibody was found to counteract the anti-nociceptive effects of gabapentin in morphine-tolerant neuropathic pain rats.

Previous studies have shown that IL-10 exerts its anti-inflammatory effects via the upregulation of HO-1 expression (Gomez-Hurtado et al. 2011). The anti-inflammatory effects of HO-1 have been demonstrated in several neuropathic and inflammatory pain models (Hervera et al. 2012). HO-1 induction by epibatidine exhibits anti-nociceptive and anti-inflammatory effects via the activation of methyllycaconitine-sensitive nAChRs (Egea et al. 2009). HO-1 mediates the anti-inflammatory effects of acute alcohol on IL-10 activation in monocytes (Milligan et al. 2005; Rittner et al. 2005). These results showed that HO-1 plays a crucial role in the host defence mechanism against inflammation; HO-1 catabolises heme and generates the antioxidants biliverdin and bilirubin, which protects against tissue injury-induced inflammation (Rosa et al. 2008). In addition, a recent study indicated that anti-IL-10 antibody dose-dependently reversed the amitriptyline-induced HO-1 expression in amitriptyline/morphine co-infused rats (Tai et al. 2009). Inhibition of HO-1 protein synthesis or activity significantly reversed the inhibitory effect of IL-10 on production of TNF-α induced by lipopolysaccharide (LPS; Lee and Chau 2002). In IL-10 knock-out mice, the expression of HO-1 was to a lower extent than in corresponding wild-type mice under 24 h post-ischemia (Perez-de-Puig et al. 2013). Treatment with HO-1 improved the local anti-nociceptive effects of morphine during chronic inflammatory and neuropathic pain in mice (Hervera et al. 2013). In our current study, we found that gabapentin/morphine co-injection significantly increased IL-10 and HO-1 expression and that it decreased the protein levels of the pro-inflammatory cytokines TNF-α, IL-1β, IL-6. In contrast, inhibition of IL-10 or HO-1 expression significantly downregulated HO-1 expression and increased TNF-α, IL-1β and IL-6 protein levels. Moreover, the HO-1 expression was transcriptionally induced by IL-6 in a time and dose-dependent manner (Tron et al. 2006). These results suggested a role of HO-1 in the anti-inflammatory effect of IL-10 in gabapentin/morphine-coinjected SNL rats. Taken together, HO-1 may be responsible for the protective effects of IL-10; however, the detail mechanisms remain undetermined.

Several studies have demonstrated that gabapentin enhanced the anti-nociceptive effect of morphine in neuropathic pain and visceral pain rats (Smiley et al. 2004; De la O-Arciniega et al. 2009). The combination of gabapentin and morphine, which were ineffective alone, produced a significant analgesic effect in an animal writhing model of pain (Meymandi and Sepehri 2008). Gabapentin alone did not produce an anti-nociceptive effect, whereas the combined treatment of morphine and gabapentin completely decreased allodynia behaviour at 30 min post-injection, an effect that persisted until 120 min in neuropathic pain induced by chronic constriction injury in rat (De la O-Arciniega et al. 2009). Moreover, the anti-nociceptive effect induced by gabapentin may be inhibiting via the release of an inflammatory mediator. Intrathecal gabapentin increased IL-10 expression and inhibited the expression of the pro-inflammatory cytokines TNF-α, IL-1β and IL-6 in a rat model of neuropathic pain (Lee et al. 2013). Our previous study found that gabapentin enhanced the morphine anti-nociceptive effect during acute and chronic morphine treatments via IL-10 and that anti-IL-10 antibody reversed the effects of gabapentin on morphine. Similarly, gabapentin was shown to diminish CX3CL1 signalling and spinal microglia activation induced by joint inflammation (Yang et al. 2012). Moreover, it has been demonstrated that the degree of neuroinflammation induced by neuropathy combined with chronic administration in nerve-injured rats was higher compared to the neuroinflammation induced by neuropathy alone (Raghavendra et al. 2002). This suggested that neuroinflammation is a common mechanism in both neuropathy-induced and chronic morphine-induced glial activation (Raghavendra et al. 2004; Liu et al. 2011). In the current study, daily anti-IL-10 and HO-1 antibody injection produced significant pro-inflammatory cytokine expression and attenuated the anti-nociceptive effect of gabapentin in morphine-tolerant neuropathic pain rats. Conversely, daily IL-10 (5 μg)/morphine injections produced a significant enhancement of the anti-nociceptive effect compared with morphine injection alone in rats. Thus, the increased pro-inflammatory cytokines expression observed in morphine-tolerant rats contributes to the development of allodynia induced by neuropathic pain. Moreover, morphine tolerance and the inhibition of pro-inflammatory cytokine expression can potentiate the anti-nociceptive effects of gabapentin on morphine. Based on our study, intrathecally administered gabapentin enhances IL-10, HO-1 expression and counteracts morphine tolerance-induced neuroinflammation and may be responsible for preventing neuropathic pain and inhibiting morphine tolerance-induced pro-inflammatory cytokine expression in the rat spinal cord. However, further studies are required to elucidate the underlying mechanism of i.t. gabapentin action and the precise mechanism by which gabapentin induces the upregulation of IL-10 and HO-1 expression.

Consequently, co-injection of gabapentin/morphine attenuated morphine tolerance in neuropathic pain rats. We propose that the effect of gabapentin may be due, at least in part, to the upregulated expression of anti-inflammatory cytokine IL-10 and a stress-induced protein HO-1 in the spinal cord, which results in the inhibited expression of pro-inflammatory cytokines TNF-α, IL-1β and IL-6 in morphine-tolerant rat spinal cords. Thus, we suggest that gabapentin may be used as an adjuvant in combination with opioids for the treatment of chronic neuropathic pain conditions and for treatment of patients who require long-term opioid administration for pain management in clinical practice. Taken together, these findings may explain why gabapentin plays an important role in the treatment of chronic neuropathic pain via systemic administration. The systemic effect and mechanisms of action of gabapentin in morphine tolerance development and neuropathic pain requires further investigation.
